# Cost-effectiveness of pharmacological and psychosocial interventions for schizophrenia

**DOI:** 10.1186/1478-7547-9-6

**Published:** 2011-05-13

**Authors:** Pudtan Phanthunane, Theo Vos, Harvey Whiteford, Melanie Bertram

**Affiliations:** 1 Setting Priorities Using Information on Cost-Effectiveness (SPICE) project, Ministry of Public Health, Nonthaburi, Thailand; 2 School of Population Health, the University of Queensland, Herston, QLD Australia; 3 Queensland Centre for Mental Health Research, The Park Centre for Mental Health, Summer Park, QLD Australia; 4 Faculty of Management and Information Sciences, Naresuan University, Phitsanulok, Thailand

## Abstract

**Background:**

Information on cost-effectiveness of interventions to treat schizophrenia can assist health policy decision making, particularly given the lack of health resources in developing countries like Thailand. This study aims to determine the optimal treatment package, including drug and non-drug interventions, for schizophrenia in Thailand.

**Methods:**

A Markov model was used to evaluate the cost-effectiveness of typical antipsychotics, generic risperidone, olanzapine, clozapine and family interventions. Health outcomes were measured in disability adjusted life years. We evaluated intervention benefit by estimating a change in disease severity, taking into account potential side effects. Intervention costs included outpatient treatment costs, hospitalization costs as well as time and travel costs of patients and families. Uncertainty was evaluated using Monte Carlo simulation. A sensitivity analysis of the expected range cost of generic risperidone was undertaken.

**Results:**

Generic risperidone is more cost-effective than typicals if it can be produced for less than 10 baht per 2 mg tablet. Risperidone was the cheapest treatment with higher drug costs offset by lower hospital costs in comparison to typicals. The most cost-effective combination of treatments was a combination of risperidone (dominant intervention). Adding family intervention has an incremental cost-effectiveness ratio of 1,900 baht/DALY with a 100% probability of a result less than a threshold for very cost-effective interventions of one times GDP or 110,000 baht per DALY. Treating the most severe one third of patients with clozapine instead of risperidone had an incremental cost-effectiveness ratio of 320,000 baht/DALY with just over 50% probability of a result below three times GDP per capita.

**Conclusions:**

There are good economic arguments to recommend generic risperidone as first line treatment in combination with family intervention. As the uncertainty interval indicates the addition of clozapine may be dominated and there are serious side effects, treating severe patients with clozapine is advisable only for patients who do not respond to risperidone and only in the presence of a stricter side effect monitoring system than currently exists.

## Background

Schizophrenia generally begins in early adulthood and causes long term mental and physical impairment [1]. It has a significant impact on individuals, families and countries in terms of both health and economic loss. In the 1999 Thai Burden of Disease and Injury study, schizophrenia was responsible for 5% of all non-fatal health loss measured in years lived with disability [2]. The direct health care costs of schizophrenia account for between 1% and 3% of total national health care expenditure worldwide [3-5]. Indirect costs related to lost productivity are estimated to be higher or at least equivalent to direct costs, in the range of 1 to 7 times the direct costs [3,5-7]. The evidence, however, indicates that Asian countries have a larger proportion of indirect costs (87% in Taiwan, 83% in Korea, and 63% in India) [5,8,9] than those reported in Western countries (47% to 70%) [3].

Thailand has limited health resources for mental disorders, including schizophrenia. The government devoted only 3.0% of health expenditure to mental health in 2008 [10]. It is therefore paramount that decision makers have access to cost-effectiveness information to prioritise allocation of resources within their budget constraints.

Medications are the standard treatment to control acute psychotic symptoms of schizophrenia. The newer 'atypical' antipsychotics have a similar effect on psychotic symptoms as first-generation 'typical' antipsychotics but cause different side effects and are much more expensive [11-13]. The key problems with using medications alone are poor adherence and a partial improvement in functional outcomes only [14]. A combination of drug and non-drug interventions is commonly recommended [15-17].

To our knowledge, there are no cost-effectiveness studies indicating what should be the first and second line drug treatments in Thailand. This study aims to provide policy makers with evidence on the optimal package of drug and non-drug interventions for schizophrenia. We undertook the current study as part of the Setting Priorities using Information on Cost-Effectiveness (SPICE) project which aimed to provide comparable cost-effectiveness results across various disease areas to assist policy makers in priority setting decision making.

## Methods

We selected interventions for analysis based on: a) efficacy and/or effectiveness in published literature; b) availability of evidence on clinical effectiveness, resource utilization and costs; c) feasibility of implementation in Thailand based on discussions with 10 local mental health experts; and d) relevance to current policy-making. Four drug interventions (typicals, risperidone, olanzapine and clozapine) and family interventions were chosen for analysis. The comparator was a hypothetical 'do nothing' scenario using generalized cost-effectiveness. This required a back-calculation from current practice to the 'partial null', i.e. a hypothetical scenario where we removed the effect of all currently implemented interventions [18,19]. An 'intervention pathway' was calculated to determine the most cost-effective mix of interventions.

**Figure 1:**
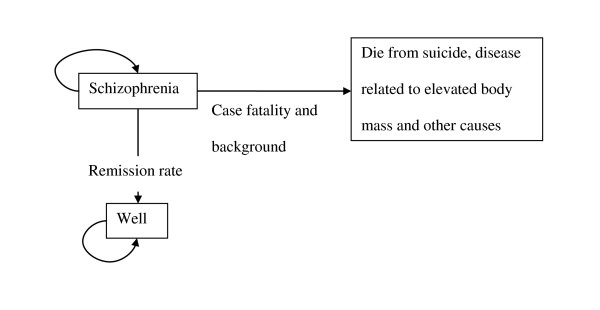
Health state diagram

Our study design was cost-effectiveness analysis using a Markov model with cycles of one year. Patients with schizophrenia in Thailand in the year 2005 were the target population. Each year patients could remain alive with schizophrenia, recover or die from disease-related or other causes (Figure 1). To reflect the chronic course of schizophrenia, costs and effects were assessed until age 80 or death. The annual transition probabilities were based on local and overseas data (See Additional file 1: Transition probability estimates).

The model was analyzed from a government costing perspective. Our survey showed that 75% of patients with schizophrenia used the universal coverage health care system [20]. However, because schizophrenia has significant impacts outside the health sector [1,3], we also considered costs to patients and families (time and travel costs). Discounting at 3% was applied to both costs and benefits to be consistent with the study of Burden of Disease in Thailand and in line with recommendations from the Panel on Cost-Effectiveness in Health and Medicine [2,21]. Our model was constructed using Microsoft Excel.

### Health effect estimations

Health outcomes from the drug and non-drug interventions were measured in disability-adjusted life years (DALYs) averted using identical methods. Intervention effect size was measured as a change in disability weight (DW) using a method described by Magnus et al [22] to translate pooled effect sizes from the international literature into a change in DW. To combine effect sizes, Hedges' g test was applied to determine the mean difference in standard deviation units including an adjustment to correct for small sample sizes [23]. Because the psychiatric literature usually reports efficacy measures based on different psychometric scales [24], we calculated the effect sizes based on the Brief Psychiatric Rating Scale (BPRS) and the Positive and Negative Syndrome Scale (PANSS) in our meta-analyses since they are the most common scales for scoring psychiatric symptoms. Our analyses relied heavily on the information on intervention effects from Cochrane systematic reviews [25-30]. The standardized effect sizes were then pooled using the random effects meta-analysis method to deal with variation around some overall average treatment effect [23]. The method used in the Australian Assessing Cost Effectiveness - Mental Health (ACE-MH) project [22,24] to transfer the pooled effect sizes into a change in disability weight was adjusted to include the spread of the Brief Psychiatric Rating Scale-Expanded (BPRS-E) scores from our survey, a cross-sectional descriptive study including 307 people with schizophrenia in Thailand (279 outpatients and 28 inpatients) [20,31]. We mapped each BPRS-E score (ranging from 24 = least severe to 134 = most severe) to the Dutch lowest and highest DWs for schizophrenia (0.21-0.98) using the methods developed by the ACE-MH Study [24,32]. Note that the lowest disability weight for schizophrenia still means a substantial loss of health reflecting the disability associated with a stigmatized illness as well as some residual symptoms. Across the survey sample we calculated the standard deviation of the BPRS scores and estimated the average health loss (in disability weight) associated with a 1 SD change in severity [20]. As the effect sizes for the impact of interventions are expressed in standard deviation units, we could then impute the average change in disability weight given an effect size for each intervention. The impact of a change in extrapyramidal (EPS) adverse effect was estimated separately and transferred into an additional change in DW. We borrowed data on the proportions of patients with moderate and severe EPS due to typicals (25%) and atypicals (13%), as well as the DW of each severity level, i.e. 0.422 and 0.453 for moderate and severe EPS, respectively from the Australian study [22] to calculate the DW change due to less EPS on atypicals. Due to lack of data we had to assume that all the atypicals induced EPS equally.

Apart from accounting for the EPS side effects in the change in DW, we also estimated the mortality and morbidity risks associated with the average weight gain associated with each drug using the potential impact fraction RiskIntegral add-in for Excel (http://www.epigear.com). The benefit of preventing suicide was taken into account for clozapine, the only antipsychotic drug with evidence of this effect [33]. The weighted average relative risk of suicide for people taking clozapine was 0.53, calculated using data from studies by Munro [34] and Harris [35]. Table 1 shows the input parameters used to estimate intervention benefits.

**Table 1:** Input parameters for estimation of health outcome for drug and non-drug interventions compared to 'do nothing'

Intervention	DW change (95% CI)	RR of suicide	Weight change (kg)	proportion of patients with moderate and high EPS
Typicals	-0.069 (-0.032, -0.101)	1	1.42	25%
Risperidone	-0.085 (-0.050, -0.116)	1	2.10	13%
Olanzapine	-0.095 (-0.060, -0.127)	1	4.15	13%
Clozapine	-0.099 (-0.067, -0.128)	0.54	4.45	13%
Family intervention^1^	-0.076 (-0.064, -0.085)	1	-	-

Data source: RR of suicide [34,35]; Weight change [56] and Proportion of patients with moderate and high EPS [22,57]

^1 ^Additional benefit to drug interventions

Due to the absence of Thai data on adherence to drug treatment for schizophrenia, we assumed adherence of 47% (« 7.4%) from a review study by Lacro et.al [36]. A study on depression treatment in Thailand found adherence of 41%, indicating a similar level of adherence within the mental health system [37]. We applied this rate for both people receiving atypical and typical antipsychotics [38,39]. We provide further details of health effect estimation via the online document (See Additional file 1: Health effect estimations)

### Cost estimations

The cost analyses comprise of: a) intervention cost; b) medical costs for treating or preventing side effects; c) cost of hospitalization; and d) time and travel cost of patients and families. All costs were adjusted to 2005 using the Consumer Price Index (CPI) based on data from the Bank of Thailand website (http://www.bot.or.th).

We estimated the annual drug cost for each of the antipsychotics by combining the unit cost for a standard daily dose with the costs of drug administration (i.e. outpatient visits). Costs of benzhexol, an anticholinergic drug that treats EPS side effects were also included. We separately measured costs of regular blood tests to prevent the fatal adverse effect of clozapine, agranulocytosis [40]. We used a 27% hospitalization rate among patients on typicals based on local data [20] while adopting the relative reduction in hospitalization rates in patients taking atypicals from Leucht et al. [41]. According to the Cochrane review study, we assumed that by comparison with no treatment typicals reduce the hospitalization rate by 50% [42].

The costs of family interventions mainly consisted of start-up costs (development and training) and ongoing costs (services, supplies, travel and salary). We assumed that the family intervention protocol would be prepared by 2 specialists employed by the project for 100 hours each and a training program would be conducted for a group of clinicians from all 17 psychiatric hospital across Thailand. One psychiatrist and 20 psychiatric nurses from each hospital would be trained. The family intervention program would consist of 10 weekly 2-hr sessions by a psychiatric nurse [43]. We assumed that each session would have 16 participants (8 patients and 8 family carers). As there is no information on the longer term outcomes of family interventions, we assumed after expert consultation that 2 booster sessions to patients and families every year, would enable the benefit of FI to be maintained over a patient's lifetime.

Time cost was assumed to be 25% of personal income per capita in Thailand in 2005 (http://www.nesdb.go.th) and travel costs of patient and families were computed using patient-reported data from our survey [20]. The cost parameters, values and data sources are described in the online supporting document (See Additional file 1: Assessment of intervention cost).

### Sensitivity analysis

One way sensitivity analysis was undertaken for (a) intervention costs including and excluding time and travel costs of patients and families; and (b) varying costs of risperidone. Generic risperidone is not yet available in Thailand. However, the Royal College of Psychiatrists of Thailand has urged the Government Pharmaceutical Organization to produce a generic version of risperidone following the expiration of its patent and it is currently in the process of Food and Drug Administration approval [44]. In this sensitivity analysis we modelled risperidone prices ranging from 4 baht (expected generic price [44]) to 50 baht. We compared risperidone to typicals in this sensitivity analysis.

Probabilistic sensitivity analysis using Monte Carlo simulation was undertaken using an Excel add-in Ersatz (http://www.epigear.com). The uncertainties in all values were considered simultaneously using appropriate distributions and relevant parameters. Additional online information provides the details of input parameters, distributions and data sources used for uncertainty analysis (See Additional file 1: Uncertainty parameters and distributions.

### Additional policy-relevant criteria

While the initial results were based on cost-effectiveness analysis, broader aspects which may be important in terms of policy, known as "second filter" criteria, were also considered. In Australian 'Assessing Cost-Effectiveness' studies 'strength of evidence', 'equity', 'feasibility', 'acceptability' and sustainability have been considered [45]. Our expert advisory group and the steering committee of the SPICE project played an important role in applying these second filter criteria and formulating policy recommendations.

## Results

Atypical antyipsychotics were not significantly more effective than typicals in reducing the severity of disease (Tables 1 and 2). Clozapine had an additional benefit of preventing suicide but greater health loss from weight gain than the other drugs (Table 2). Family intervention had additional health benefits when combined with drug treatment.

**Table 2:** Health effects of typical and atypical antipsychotic compared to 'do nothing' over the lifetime people with schizophrenia in the 2005 Thai population

Intervention	DALYs averted			
	Severity of disease	Weight gain	Mortality due to suicide	Total (95% CI)
Typicals	450,000	-5,800	-	440,000 (290,000, 610,000)
Risperidone	540,000	-9,100	-	530,000 (370,000, 710,000)
Olanzapine	580,000	-14,000	-	570,000 (390,000, 770,000)
Clozapine	620,000	-15,000	20,000	630,000 (440,000, 830,000)
FI+Risperidone^1^	920,000	-9,100	-	910,000 (640,000, 1,100,000)

^1 ^FI = family intervention; results are for risperidone together with FI.

At a cost of 4 baht per 2 mg tablet, generic risperidone would be the preferred drug treatment due to lower hospital costs despite higher drug costs (Table 3). While all atypicals have lower hospital costs than typicals, the drug cost of risperidone, only, was offset by hospital saving. The annual cost of clozapine treatment per individual was about twice that for typicals or risperidone, mostly because of the higher cost of monitoring side effects and the associated time costs of patients. Olanzapine was the most costly drug, approximately 7 times higher than typicals and risperidone. Giving risperidone with family interventions was more expensive than risperidone alone by 4,000 baht in the first year due to higher patient and family costs.

**Table 3:** Costs of interventions analyzed per individual receiving the intervention in the first year

	Cost (baht/year)				
Drug intervention	Intervention	Side effects	Hospitalization	Time cost	Total (95% CI)
Typicals	3,300	400	8,100	1,300	13,000 (11,000, 15,000)
Risperidone	4,300	250	5,200	1,300	11,000 (9,500, 13,000)
Olanzapine	81,000	160	5,200	1,300	88,000 (84,000, 93,000)
Clozapine^1^	4,600	3,800	5,200	6,900	21,000 (19,000, 22,000)

FI^2^+Risperidone	6,300	-	4,500	4,400	15,000 (13,000, 17,000)

Notes: all estimates are rounded to two significant digits

^1 ^Clozapine is associated with rare but serious blood disorders and hence frequent blood tests are required.

^2 ^FI = family intervention; cost of FI shown is that of the more intensive first year.

By comparison with 'do nothing', most of the selected interventions, with the exception of clozapine and olanzapine, were cost-saving (Table 4). The cost-effectiveness ratio of clozapine (12,000 baht/DALY averted) was below Thailand GDP per capita of 110,000 baht which is considered the threshold for very cost-effective health interventions (http://www.dcp2.org). When we included costs of patient and family time in the analyses, all of the interventions were no longer dominant but their cost-effectiveness ratios (except for clozapine and olanzapine) still fell well below the threshold of 110,000 baht. Olanzapine (1,000,000 baht/DALY averted) has an unfavourable cost-effectiveness ratio due to its high cost for at most only a small additional benefit over other drug treatments.

**Table 4:** Average cost-effectiveness ratios of interventions

Intervention	Average cost-effectiveness ratio			
	Tx^1^	Tx+Hosp^2^	Tx+Time^3^	Total
Typical	64,000	Dominant^4^	87,000	Dominant
Risperidone	66,000	Dominant	85,000	Dominant
Olanzapine	1,100,000	980,000	1,100,000	1,000,000 (730,000; 1,400,000)
Clozapine	80,000	Dominant	130,000	12,000 (Dominant; 38,000)
FI^5 ^+ Risperidone	42,000	Dominant	57,000	Dominant
Risperidone (2/3) & Clozapine (1/3)^6 ^+ FI	45,000	Dominant	67,000	Dominant

^1 ^Tx = average cost-effectiveness ratio taking into account treatment costs only

^2 ^Tx+Hosp = average cost-effectiveness ratio taking into account treatment and hospital costs

^3 ^Tx+Time = average cost-effectiveness ratio taking into account treatment and time and travel costs

^4 ^Dominant = more effective and less costly than comparator \

^5 ^FI = family intervention

^6 ^Giving clozapine to one-third of patients with high severity

**Figure 2:**
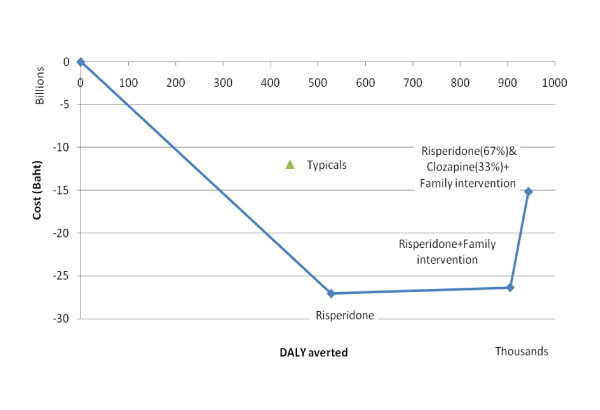
Ideal mix of schizophrenia interventions based on their cost-effectiveness ratio in Thailand

The ideal intervention package would start with generic risperidone, assuming the cost is 4 baht per 2 mg tablet (Figure 2 and Table 5). The next step would be to add family interventions to risperidone with significant health gain at a small net cost (ICER 1,900 baht/DALY; 95%CI: "dominant", 18,000; 100% probability of a result below the one times GDP per capita). Providing clozapine instead of risperidone to the most severe one-third of patients has an ICER of 320,000 baht/DALY (95% CI: 26,000, "dominated"; 51% probability of a result below the three times GDP per capita threshold of 330,000 baht/DALY for cost-effective interventions). We assumed that patients who had a score greater than 40 on the BPRS (this would include about one-third of patients in our survey) would be eligible for clozapine [20,31]. "Dominated" denotes an intervention more costly and less effective than the comparator, while "dominant" means the intervention is more effective and less costly than the comparator.

**Table 5:** Incremental cost-effectiveness ratios of optimal package of interventions

Intervention(s)	Incremental cost-effectiveness ratio	Probability of ICER below	
		1 × GDP	3 × GDP
Risperidone	Dominant	100%	100%
Add Family intervention	1,900	100%	100%
Add clozapine to most severe one third replacing risperidone	320,000	38%	51%

**Figure 3:**
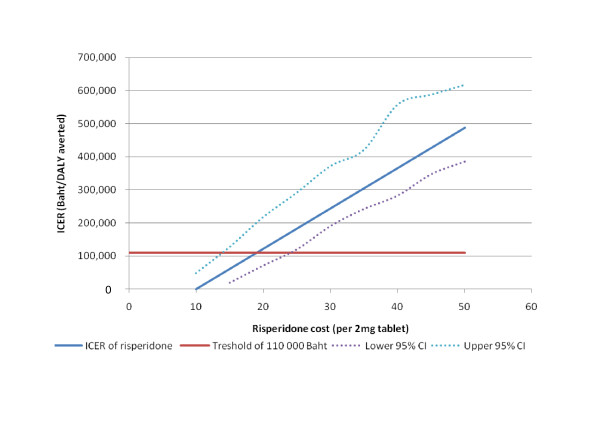
Incremental cost effectiveness analysis results using the range of risperidone costs by comparison with typicals

Analysis of generic risperidone was based on an "expected cost". The actual cost for generic risperidone could have a significant impact on the cost-effectiveness, so we examined potential values in a one-way sensitivity analysis (Figure 3). If generic risperidone costs less than 10 baht per 2 mg tablet, it is dominant over typicals. Up to a cost of 19 baht (95% uncertainty interval 15-25 baht) replacing typicals by risperidone would still be considered a very cost-effective intervention against a threshold of 110,000 baht.

Based on the CEA results, the recommended intervention package would be the combination between risperidone, clozapine and family interventions. Providing clozapine for highly severe patients cannot be recommended until it is ensured that its serious side effect of agranulocytosis is prevented: patients receive periodic blood test during this treatment (Table 6).

**Table 6:** Considerations of additional policy-relevant criteria

Criteria	Intervention package for schizophrenia
Level of evidence:	• We have relied heavily on evidence provided in Cochrane systematic reviews and used their inclusion criteria when adding more recent trials. • There is sufficient data for drug interventions while few trials are available for psychosocial intervention. Most trials were conducted in Western countries and doubts remain about the applicability of these findings to Thailand. A number of further methodological problems were identified in this trial literature: the varying choice of comparator in the evaluation of psychosocial interventions; use of high doses of typicals as comparator in trials of newer generation drugs; potential publication bias; and short durations of follow-up
Equity:	• Generic risperidone is recommended as the first line drug treatment and should be included in the national essential drug list so that it can be prescribed equitably to all Thai patients. • Lack of mental health resources and higher travel costs for patients and families may make family interventions less available or affordable in rural and remote areas. Poor households may have less access to this intervention.
Feasibility:	• Increased prescribing of clozapine in Thailand would be advisable only if health care units can maintain monthly blood testing. This would require additional support from the government to help patients and families with the travel cost. • The availability of mental health resources psychiatric nurses or psychologists, training programs and accessibility of health care units are of concern. • Maintaining the benefit of family interventions annually is possibly more difficult than doing it in the first year for a number of reasons: (a) the patients migrate to other areas; (b) the families have no time available during the program period; (c) lack of long term budget to support the program; and (d) the trained nurses change workplace or quit the careers.
Acceptability:	• The major effectiveness measurement of this study is symptom reduction based on clinical perspectives. In fact, the patient and families might have different views. This measurement by clinicians may be too limited from the point of view of patients and families who are interested in general wellbeing and productivity gains, for example. However, recent studies [58-60] found that psychiatric symptoms are the best independent predictors of these broader outcomes of schizophrenia (e.g. objective and subjective quality of life and social skill functions). • Some policy makers and clinicians may be reluctant to use clozapine due to its rare but fatal side effect. Although a recent long-term follow-up study in Finland found a lower mortality rate among people with clozapine than those with other antipsychotics [61], less intensive monitoring for those on clozapine treatment in Thailand than Finland means these low mortality rates are unlikely to be achieved [47,61]. • Self-stigma of people with schizophrenia and their families could be an obstacle to involvement in a psychosocial intervention program [48].
Sustainability:	• In order to maintain lifetime benefits due to family interventions, a long-term public funding is required from the government.

## Discussion

The three main findings from this study are (a) generic risperidone should be used as the first line drug treatment for schizophrenia if the cost is less than 10 baht per 2 mg tablet; (b) combining risperidone with family interventions will substantially increase health gain with lower hospitalization cost; and (c) clozapine could be a second line medication for patients with high severity who fail to respond to risperidone.

The key recommendation of this study is that risperidone should be included in the national drug list while olanzapine should not (Table 6). Clozapine should be reserved for severe patients who do not respond to risperidone as it may have superior benefits in treatment-resistant schizophrenia and in preventing suicidal behaviour [33,46]. However, the poor compliance with the Clozapine Patient Monitoring Service program in Thailand [47] means that clozapine cannot be recommended until monitoring has improved.

The stigma of schizophrenia could be a significant barrier to family interventions [48]. More importantly, due to a lack of mental health resources and high travel costs, poor households and people living in rural areas may have limited access. A new stream of long term government funding would be required to provide family interventions across the country and make these services accessible long-term.

This study has several limitations. First, the studies included in our meta-analyses were generally from Western countries not Thailand or other Asian countries. Second, the course of disease varies considerably between patients. Modelling the average cost and impact on the average case of disease could lead to erroneous conclusions [49]. The alternative is to use a microsimulation approach; however, that would require more clinical and epidemiological information on individuals than we had available. Third, the methods used for translating the effect sizes into a change in DW need the assumption that the effect sizes from trials can be directly applied to general health status estimated using a generic measurement. Normally, generic measurements are less relevant to schizophrenia's symptoms by comparison with specific ones as BPRS used in this study. However, this is the only method allowing us to clarify DWs in patients with different treatments. An advantage of using this method is that we could include Thai data on the individual patients into the analysis. We would content that it is not necessarily so that departure from the assumed linear relationship between symptom change and disability change would lead to over-estimation of health benefits in DALYs. Fourth, the assumption that patients remain on the same treatment throughout the course of their illness is in contrast to the common clinical practice of switching patients from one treatment to another treatment based on individual medication responses [50]. However, a large double-blinded randomized clinical trial in the US suggested no difference in rates of improvement between patients with atypical antipsychotics switching to a new medication and patients staying on their initial treatment [51]. Finally, we have not incorporated the costs of treating diseases related to increased body weight due to unavailability of local disease costing data. Because there is only a small difference in weight gain between typicals and risperidone, this limitation is unlikely to affect the findings.

Our study also has several major strengths. First, the information used for assessing costs and effectiveness of each intervention is documented and transparent, and from Thai sources wherever possible. Second, to our knowledge, there is no previous study including time and travel costs in the cost estimations. Our findings show that these are a significant burden on patients and families, particularly for clozapine and family interventions. Third, we clearly address the issue of uncertainty in the data, presenting our results as a range within which the value is expected to fall. Fourth, we have had regular contact with local policy makers over the five-year span of our work. As a result of this, the food and drug administration in Thailand has already allocated 40 million baht to add generic risperidone to the national essential drug list.

This is the first study of its kind in Thailand. Studies elsewhere have modeled cost-effectiveness of different atypical antipsychotics, with fewer studies addressing both drug and non-drug interventions. Our results are in line with previous research. A study conducted in Slovenia which measured effectiveness as the percentage of patients in remission found that risperidone was more cost-effective than haloperidone and olanzapine for schizophrenia [52]. Studies in Canada, Brazil and Australia consistently found that using risperidone rather than olanzapine would reduce the medical costs of schizophrenia [22,53,54]. In contrast, a number of studies using the method developed by the WHO-CHOICE project suggested using typical antipsychotics in combination with psychosocial interventions [55]. However, the authors discussed that if generic forms of atypical medication would become available, the findings could change [55] as our sensitivity analysis indicates. Costs associated with drug treatments are a major driver of the results of cost-effectiveness analysis and limit the applicability of our conclusions to other countries.

A combination of psychosocial interventions and medications has been highly recommended as a successful treatment package for schizophrenia [16]. Our results confirm that this package not only provides more health gain to patients, but also could help the government to reduce hospitalization cost by as much as 40% compared to typicals (Table 3). The hospitalization cost is generally considered to be the most expensive component of direct costs [3,5,8,9].

## Conclusions

The cost of medications is the most important factor in the cost-effectiveness of antipsychotic interventions for schizophrenia. Atypicals are more cost-effective than typicals if their generic versions can be produced at a cost level achieved for many other generic medications in Thailand. Family intervention is an additional cost-effective option to help not only improve disease severity, but also reduce hospitalizations thus lessening the economic burden on the government.

## Competing interests

The authors declare that they have no competing interests.

## Authors' contributions

PP, TV and HW participated in the study design and data collection. PP, TV and MB conducted the data analysis and contributed to design of the modelling strategy. PP wrote the first draft of the manuscript. All authors have been involved in reviewing the manuscript and have given final approval of this manuscript version

## Supplementary Material

Additional file 1 **Estimations of transition probabilities, health outcomes and intervention costs. 1) Transition probability estimates - **This file explains a Markov cohort model with 3 health states: alive with schizophrenia, alive without schizophrenia and dead due to suicide, increased body weight or other causes. **2) Health effect estimations - **This file describes measurement of the health benefit as a change in severity of disease calculated as a change in disability weight. Additionally, it provides details on estimation of the mortality and morbidity risks associated with weight gain. **3) Uncertainty parameters and distributions - **This file gives information on uncertainty parameters, distributions used and data sources. **4) Assessment of intervention cost - **This file gives details on cost estimates including cost elements, cost value and data sources. Click here for file
