# Supplementary material for: Cost-effectiveness of pharmacological and psychosocial interventions for schizophrenia
Source: Cost Eff Resour Alloc. 2011 May 13;9:6. doi: 10.1186/1478-7547-9-6 (PMC3120770; doi:10.1186/1478-7547-9-6)
Supplement: Additional file 1 — Estimations of transition probabilities, health outcomes and intervention costs. 1) Transition probability estimates - This file explains a Markov cohort model with 3 health states: alive with schizophrenia, alive without schizophrenia and dead due to suicide, increased body weight or other causes. 2) Health effect estimations - This file describes measurement of the health benefit as a change in severity of disease calculated as a change in disability weight. Additionally, it provides details on estimation of the mortality and morbidity risks associated with weight gain. 3) Uncertainty parameters and distributions - This file gives information on uncertainty parameters, distributions used and data sources. 4) Assessment of intervention cost - This file gives details on cost estimates including cost elements, cost value and data sources. [file 1478-7547-9-6-S1.DOC]

**Additional file**

This additional material to the article “Cost-effectiveness of pharmacological and psychosocial interventions for schizophrenia” provides additional information on model structures, model assumptions, and model variables as well as the estimations of costs and benefits.

1. **Transition probability estimates**

We developed a Markov cohort model with 3 health states: alive with schizophrenia, alive without schizophrenia and dead due to suicide, increased body weight or other causes. The annual transition probabilities were drawn from local and overseas data.

The national prevalence of schizophrenia was estimated from a community mental health survey and a count of admissions in a hospital dataset [1]. A pooled annual remission rate of 1.37% was derived from a meta-analysis of 12 studies [2] and a relative risk of mortality of 2.58 in patients with schizophrenia from a recent meta analysis [3]. An Incidence-Prevalence-Mortality model, DisMod [4-5], was used to derive the desired model parameters of incidence and case fatality. In DisMod, we imposed an age pattern for RR of mortality from two studies [6-7] on the overall estimate of RR.

Overall mortality rates came from the mortality component of the SPICE project conducted between 2005 and 2008 [8-9]. We estimated a pooled annual risk of suicide in schizophrenia of 0.0066, calculated based on 61 studies reported in a systematic review by Palmer [10] and then imposed the age-sex pattern of overall suicide mortality in Thailand [8-9]. We modeled that an increased body mass index (BMI) from some schizophrenia treatments was linked to a greater risk of death from ischemic heart disease, ischemic stroke, hypertensive disease, diabetes, colon and rectum, breast cancer, corpus uteri cancer and osteoarthritis based on meta-analyses carried out for the WHO comparative risk assessment study [11].

1. **Health effect estimations**

We measured the health benefit as a change in severity of disease calculated as a change in disability weight, contributing to an overall outcome measure of disability adjusted life years (DALYs) averted [12].

The pooled effect size, i.e. the standardized mean difference in continuous outcomes between two groups, based on randomized control trials up to 2007 was calculated (Table 1).

**Table 1** Effect sizes and their Hedges’ g scales of studies included in our meta analyses

| **Study** | **Measure** | **Treatment** | | | **Control** | | **Hedges’ g scale** | | |
| --- | --- | --- | --- | --- | --- | --- | --- | --- | --- |
|  |  | **Sample size** | | **Mean (SD)** | **Sample size** | **Mean (SD)** | **Mean** | | **SE** |
| **1) Typicals vs. placebo** | | | | | | | | | |
| Borison, 1991 [13] | BPRS | 9 | | 46 (5) | 10 | 51 (6) | | 0.70 | 0.48 |
| Tetreault, 1969 [13] | BPRS | 15 | | 41 (10) | 15 | 49 (10) | | 0.75 | 0.38 |
| Bechelli, 1983 [13] | BPRS | 29 | | 25 (8) | 23 | 36 (12) | | 1.20 | 0.30 |
| Jarn, 1997 [13] | BPRS | 12 | | 36 (11) | 8 | 47 (15) | | 0.88 | 0.48 |
| **Pooled effect size = 0.945 (95%CI: 0.57, 1.33; p-value = 0.000)** | | | | | | | | | |
| **2) Risperidone vs. typicals** | | | | | | | | | |
| Blin, 1996 [14] | BPRS | 21 | | -26 (16) | 20 | -17 (16) | | 0.59 | 0.32 |
|  | PANSS | 21 | | -45(27) | 20 | -27 (28) | |  |  |
| Chouinard, 1993 [14] | BPRS | 92 | | -9 (17) | 21 | -5 (18) | | 0.21 | 0.24 |
|  | PANSS | 92 | | -15 (27) | 21 | -9 (27) | |  |  |
| Marder, 1994 [14] | BPRS | 250 | | -6 (14) | 64 | -3 (12) | | 0.25 | 0.14 |
|  | PANSS | 250 | | -10 (23) | 64 | -4 (19) | |  |  |
| Liu | PANSS | 19 | | -5 (8) | 19 | -5 (8) | | 0.00 | 0.32 |
| Peuskens, 1995 [14] | BPRS | 1129 | | -9 (13) | 223 | -8 (12) | | 0.05 | 0.07 |
|  | PANSS | 1129 | | -16 (23) | 223 | -15 (22) | |  |  |
| Keefe, 2006 [15] | PANSS | 158 | | -10 (16) | 97 | -8 (16) | | 0.12 | 0.13 |
| **Pooled effect size = 0.116 (95%CI: 0.008, 0.223; p-value = 0.035)** | | | | | | | | | |
| **3) Olanzapine vs. typicals** | | | | | | | | | |
| Bernardo, 2001 [16] | BPRS | 14 | | 37 (13) | 13 | 34 (12) | | -0.20 | 0.39 |
|  | PANSS | 14 | | 68 (23) | 13 | 63 (21) | |  |  |
| Ishigooka, 2001 [16] | BPRS | 81 | | 40(14) | 80 | 40 (13) | | -0.02 | 0.16 |
|  | PANSS | 80 | | 78 (25) | 78 | 76 (23) | |  |  |
| Chang, 2003 [16] | BPRS | 31 | | 33 (7) | 27 | 27 (9) | | -0.74 | 0.28 |
| Conley, 1998 [16] | BPRS | 42 | | 54 (14) | 39 | 57 (12) | | 0.21 | 0.22 |
| Esel, 2001 [16] | BPRS | 15 | | 24 (5) | 14 | 25 (5) | | 0.06 | 0.37 |
| Loza, 1999 [16] | BPRS | 27 | | 35 (16) | 14 | 44 (13) | | 0.63 | 0.34 |
|  | PANSS | 27 | | 97 (20) | 14 | 111 (18) | |  |  |
| Basley, 1997[16] | PANSS | 75 | | 79 (29) | 79 | 85 (26) | | 0.23 | 0.16 |
| Dehann, 2003 [16] | PANSS | 9 | | 59 (22) | 11 | 61(21) | | 0.07 | 0.45 |
| HGCQ, 2000 [16] | PANSS | 20 | | 68 (31) | 10 | 73 (31) | | 0.17 | 0.39 |
| HGDV, 1999 [16] | PANSS | 27 | | 45 (13) | 12 | 69 (23) | | 1.47 | 0.39 |
| Tollefson, 1997 [16] | PANSS | 1312 | | 72 (24) | 636 | 79 (23) | | 0.28 | 0.05 |
| Barak, 2002 [16] | BPRS | 10 | | 65 (19) | 10 | 74 (24) | | 0.58 | 0.46 |
| HGCJ, 1998 [16] | PANSS | 16 | | 57 (18) | 14 | 78 (24) | | 1.00 | 0.39 |
| HGCU, 1998 [16] | PANSS | 24 | | 62 (19) | 28 | 70 (24) | | 0.37 | 0.28 |
| Jakovlijevic, 1999 [16] | PANSS | 27 | | 57 (24) | 27 | 77 (33) | | 0.70 | 0.28 |
| Lima, 2003 [16] | PANSS | 84 | | 66 (22) | 72 | 71 (26) | | 0.22 | 0.16 |
| **Pooled effect size = 0.263 (95%CI: 0.084, 0.441; p-value = 0.004)** | | | | | | | | | |
| **4) Clozapine vs. typicals** | | | | | | | | | |
| China, 1989 [17] | BPRS | 17 | | 28 (12) | 20 | 25 (9) | | -0.28 | 0.33 |
| China, 1994 [17] | BPRS | 17 | | 30 (8) | 16 | 28 (9) | | -0.22 | 0.35 |
| Europe, 1984 [17] | BPRS | 39 | | 49 (14) | 40 | 61 (18) | | 0.74 | 0.23 |
| Germany, 1989 [17] | BPRS | 15 | | 36 (6) | 15 | 44 (11) | | 0.88 | 0.38 |
| Germany, 1994 [17] | BPRS | 37 | | 38 (18) | 45 | 38 (16) | | 0.00 | 0.28 |
| Japan, 1971 [17] | BPRS | 47 | | 37 (11) | 41 | 40 (14) | | 0.25 | 0.21 |
| Taiwan, 1997 [17] | BPRS | 19 | | 45 (12) | 19 | 52 (10) | | 0.62 | 0.33 |
| USA, 1979 [17] | BPRS | 14 | | 40 (14) | 12 | 55 (17) | | 0.91 | 0.42 |
| USA, 1987 [17] | BPRS | 62 | | 36 (14) | 63 | 40 (14) | | 0.33 | 0.18 |
| USA, 1988 [17] | BPRS | 126 | | 45 (13) | 139 | 56 (12) | | 0.84 | 0.13 |
| USA, 1996 [17] | BPRS | 10 | | 53 (13) | 11 | 65 (18) | | 0.74 | 0.46 |
| USA, 1998 [17] | BPRS | 38 | | 36 (11) | 37 | 37 (9) | | 0.14 | 0.23 |
| USA, 1994 [17] | BPRS | 29 | | 19 (12) | 23 | 18 (12) | | -0.07 | 0.28 |
| USA, 1997 [17] | PANSS | 122 | | 75 (15) | 113 | 82 (15) | | 0.47 | 0.13 |
| **Pooled effect size = 0.384 (95%CI: 0.181, 0.586; p-value = 0.000)** | | | | | | | | | |
| **5) Family interventions vs. standard care** | | | | | | | | | |
| Barrowclough, 2001 [18] | Symptoms^1^ | | 15 | 52 (14) | 14 | 59 (15) | 0.60 | | 0.37 |
|  | Function-ing^2^ | | 17 | -108 (8) | 15 | -101 (10) |  | |  |
| Xiong, 1994 [18] | Symptoms^1,3^ | | 29 | 4 (5) | 25 | 8 (7) | 0.62 | | 0.28 |
|  | Symptoms^1,4^ | | 30 | 7 (7) | 24 | 12 (7) |  | |  |
| Merinder, 1999 [18] | Symptoms^1^ | | 22 | -3 (5) | 18 | -0.3 (7) | 0.36 | | 0.32 |
| Femandez, 1998 [18] | Symptoms^1^ | | 20 | 24 (5) | 15 | 31 (10) | 1.03 | | 0.37 |
|  | Function-ing^2^ | | 20 | -124 (15) | 15 | -100 (27) |  | |  |
| Left, 2001 [18] | Function-ing^2^ | | 12 | -94 (13) | 11 | -97 (14) | -0.16 | | 0.42 |
| Li & Arthur, 2005 [19] | Symptoms^1^ | | 46 | 23 (4) | 55 | 27 (7) | 0.63 | | 0.20 |
| **Pooled effect size = 0.559 (95%CI: 0.314, 0.805)** | | | | | | | | | |

^1)^ High=poor

^2)^ High=good

^3)^ Using PANSS

^4)^ Using BPRS

***Weight gain side effects***

We estimated the mortality and morbidity risks associated with weight gain using the potential impact fraction (PIF) measurement. This was implemented using the RiskIntegral software ([www.epigear.com](http://www.epigear.com/)). PIF is a measure estimating the proportional change in disease burden resulting from a change in the exposure to a risk factor, i.e. high BMI in our case [11]. The relative risk for 1 BMI unit increase was assumed to be normally distributed around its logged value. For this calculation the PIF equation is [11]:

PIF$=\frac{\int_{\text{x}=0}^{\text{m}} \text{RR(x)P(x)dx}-\int_{\text{x}=0}^{\text{m}} \text{RR(x)P'(x)dx}}{\int_{\text{x}=0}^{\text{m}} \text{RR(x)P(x)dx}}$

where RR(x) is the risk function, P(x) is the baseline risk factor distribution, P’(x) is the risk factor distribution after the intervention, and 0 and m are the integration limits.

Mean BMI and standard deviation by age and sex were calculated using the Third Thai National Health Examination Survey (NHES-3) database [20]. Similar age-sex patterns of relative risks (RR) for a 1 kg/m^2^ BMI increase to those used for the Comparative Risk Assessment (CRA) project were implemented in this study [11]. Mean weight gain of 4.45 kg under treatment with clozapine, 4.15 kg for Olanzapine, 2.10 kg for risperidone and 1.42 kg for typicals were taken from a meta-analysis [21]. The unit change in kilograms was transferred to the BMI equivalent using the average height of male and female Thais from the NHES-3 by age group. We then multiplied the PIF attributed to each related disease by age and sex with the mortality rate and amount of disability per year.

1. **Uncertainty parameters and distributions**

**Table 2 Uncertainty parameters, distributions and sources**

| **Parameter** | **Uncertainty distribution (Mean, Standard error)** | **Source** |
| --- | --- | --- |
| Remission rate | Lognormal (0.0137, 0.0022) | [2] |
| Effect size -risperidone vs typicals^1^ | Normal (0.116, 0.055) | [14-15] |
| Effect size -olanzapine vs typicals^1^ | Normal (0.233,0.089) | [16] |
| Effect size -clozapine vs typicals^1^ | Normal (0.384, 0.103) | [17] |
| Effect size -Typical vs placebo | Normal (0.948,0.195) | [13] |
| Effect size -family intervention vs standard care^1,2^ | Normal (0.561, 0.481) | [18-19] |
| Adherence | Lognormal (0.473,0.074) | [22] |
| People with typicals, no EPS | Dirichlet (657,876) | [23-24] |
| People with typicals, moderate EPS | Dirichlet (131,876) | [23-24] |
| People with typicals, severe EPS | Dirichlet (88,876) | [23-24] |
| People with atypicals, no EPS | Dirichlet (90,104) | [23-24] |
| People with atypicals, moderate EPS | Dirichlet (7,104) | [23-24] |
| People with atypicals, severe EPS | Dirichlet (6,104) | [23-24] |
| Daily dose used-risperidone | Beta (2.27,3.69) | [25] |
| Daily dose used-olanzapine | Beta (11.43,5.36) | [25] |
| Daily dose used-clozapine | Beta (150.34,97.44) | [25] |
| Daily dose used-chlorpromazine | Beta (76.18,49.24) | [25] |
| Daily dose used-perphenazine | Beta (16.3,10.74) | [25] |
| Daily dose used-haloperidone | Beta (9.5,8.59) | [25] |
| Daily dose used-fluphenazine | Beta (8.49,8.49) | [25] |
| Daily dose used-trifuloperazine | Beta (16.74,16.74) | [25] |
| Mean BMI-male 30-44 | Lognormal (23.5,3.9) | NHES III, 2004 |
| Mean BMI-male 45-59 | Lognormal (23.8,3.9) | NHES III, 2004 |
| Mean BMI-male 60-69 | Lognormal (22.8,3.8) | NHES III, 2004 |
| Mean BMI-male 70-79 | Lognormal (21.8,3.6) | NHES III, 2004 |
| Mean BMI-male 80+ | Lognormal (20.8,3.6) | NHES III, 2004 |
| Mean BMI-female 30-44 | Lognormal (24.7,4.5) | NHES III, 2004 |
| Mean BMI-female 45-59 | Lognormal (25.5,4.4) | NHES III, 2004 |
| Mean BMI-female 60-69 | Lognormal (24.2,4.5) | NHES III, 2004 |
| Mean BMI-female 70-79 | Lognormal (22.9,4.5) | NHES III, 2004 |
| Mean BMI-female 80+ | Lognormal (21.2,4.3) | NHES III, 2004 |
| RR^3^ of ischemic heart disease-male 36-44 | Normal (1.12,0.03^4^) | [11] |
| RR^3^ of ischemic heart disease-male 45-59 | Normal (1.10,0.01^4^) | [11] |
| RR^3^ of ischemic heart disease-male 60-69 | Normal (1.06,0.02^4^) | [11] |
| RR^3^ of ischemic heart disease-male 70-79 | Normal (1.04,0.01^4^) | [11] |
| RR^3^ of ischemic heart disease-male 80+ | Normal (1.02,0.01^4^) | [11] |
| RR^3^ of hypertensive disease-male 45-59 | Normal (1.06,0.03^4^) | [11] |
| RR^3^ of hypertensive disease-male 60-69 | Normal (1.16,0.05^4^) | [11] |
| RR^3^ of hypertensive disease-male 70-79 | Normal (1.12,0.04^4^) | [11] |
| RR^3^ of hypertensive disease-male 80+ | Normal (1.06,0.02^4^) | [11] |
| RR^3^ of ischemic stroke-male 36-44 | Normal (1.14,0.04^4^) | [11] |
| RR^3^ of ischemic stroke-male 45-59 | Normal (1.10,0.03^4^) | [11] |
| RR^3^ of ischemic stroke-male 60-69 | Normal (1.08,0.02^4^) | [11] |
| RR^3^ of ischemic stroke-male 70-79 | Normal (1.05,0.02^4^) | [11] |
| RR^3^ of ischemic stroke-male 80+ | Normal (1.03,0.01^4^) | [11] |
| RR^3^ of diabetes mellitus-male 30-59 | Normal (1.19,0.06^4^) | [11] |
| RR^3^ of diabetes mellitus-male 60-69 | Normal (1.14,0.04^4^) | [11] |
| RR^3^ of diabetes mellitus-male 70-80+ | Normal (1.10,0.03^4^) | [11] |
| RR^3^ of osteoarthritis-male 30-80+ | Normal (1.04,0.01^4^) | [11] |
| RR^3^ of colon and rectum cancer-male 30-80+ | Normal (1.03,0.01^4^) | [11] |
| RR^3^ of ischemic heart disease-female 36-44 | Normal (1.12,0.03^4^) | [11] |
| RR^3^ of ischemic heart disease-female 45-59 | Normal (1.10,0.01^4^) | [11] |
| RR^3^ of ischemic heart disease-female 60-69 | Normal (1.06 0.02^4^) | [11] |
| RR^3^ of ischemic heart disease-female 70-79 | Normal (1.04,0.01^4^) | [11] |
| RR^3^ of ischemic heart disease-female 80+ | Normal (1.02,0.01^4^) | [11] |
| RR^3^ of hypertensive disease-female 45-59 | Normal (1.09,0.03^4^) | [11] |
| RR^3^ of hypertensive disease-female 60-69 | Normal (1.16,0.05^4^) | [11] |
| RR^3^ of hypertensive disease-female 70-79 | Normal (1.12,0.04^4^) | [11] |
| RR^3^ of hypertensive disease-female 80+ | Normal (1.06,0.02^4^) | [11] |
| RR^3^ of ischemic stroke-female 36-44 | Normal (1.14,0.04^4^) | [11] |
| RR^3^ of ischemic stroke-female 45-59 | Normal (1.10,0.03^4^) | [11] |
| RR^3^ of ischemic stroke-female 60-69 | Normal (1.08,0.02^4^) | [11] |
| RR^3^ of ischemic stroke-female 70-79 | Normal (1.05,0.02^4^) | [11] |
| RR^3^ of ischemic stroke-female 80+ | Normal (1.03,0.01^4^) | [11] |
| RR^3^ of diabetes mellitus-female 36-44 | Normal (1.19,0.06^4^) | [11] |
| RR^3^ of diabetes mellitus-female 45-69 | Normal (1.14,0.04^4^) | [11] |
| RR^3^ of diabetes mellitus-female 70-80+ | Normal (1.10,0.03^4^) | [11] |
| RR^3^ of osteoarthritis-female 36-80+ | Normal (1.04,0.82^4^) | [11] |
| RR^3^ of breast cancer-female 36-80+ | Normal (1.03,0.01^4^) | [11] |
| RR^3^ of colon and rectum cancer-female 36-80+ | Normal (1.03,0.01^4^) | [11] |
| RR^3^ of corpus uteri cancer-female 36-80+ | Normal (1.12,0.01^4^) | [11] |
| Weight change (kg)-clozapine | Normal (4.45,0.730) | [21] |
| Weight change (kg)-olanzapine | Normal (4.15,0.168) | [21] |
| Weight change (kg)-risperidone | Normal (2.1,0.209) | [21] |
| Weight change (kg)-typicals | Normal (1.42,0.468) | [21] |
| RR of EPS for each atypical vs haloperidone |  |  |
| Clozapine | Normal (0.03,0.88) | [26] |
| Olanzapine | Normal (0.30,0.51) | [26] |
| Risperidone | Normal (0.52,0.72) | [26] |
| Cost of professionals time | Triangle (32,147, 53,579) | [27] |
| Cost of non-professionals time | Triangle (10,571, 17, 619) | [27] |
| RR of relapse for atypicals vs. typicals | Normal (0.65, 0.07) | [28] |
| Hospitalization rate among people with typicals | Beta (57,204)^5^ | [25] |
| RR of suicide-clozapine | Lognormal (0.30,0.54) | [29-30] |

Note: Lognormal (mean, standard error); Normal (mean, standard error); Dirichlet (cases, sample size); Beta (mean, standard deviation); Triangle (lower limit, point estimate, upper limit)

^1^ The effect size of each atypical (and family intervention) versus typicals were combined with that of typicals versus placebo to determine the effect of each intervention versus ‘do nothing’, comparator used in this study

^2^ Standard care is usual level of care that involved drug interventions

^3^ Relative risk for 1 BMI unit increase

^4^ SE(ln(RR))

^5^ Beta (cases, sample size)

1. **Assessment of intervention cost**

We analyzed intervention costs as the combination of administration cost, intervention cost, medical costs for treating or preventing side effects, cost of hospitalization, and time and travel cost of patients and families (Table 3).

**Table 3 Detailed cost estimates including cost elements, cost value and data sources**

| **Cost element** | | **Value** | **Data source** |
| --- | --- | --- | --- |
| **Administrative cost** | | | |
| Outpatient visit | 662 baht /visit | | [31] |
| Inpatient visit  Outpatient visits  Admissions | 29, 000 baht /admission  4 visit/year  1 admission/year | | [31]  [31]  [31] |
| **Dosage used** | | | |
| Risperidone | | 3/day | [25] |
| Olanzapine | | 11 mg/day | [25] |
| Clozapine | | 150 mg/day | [25] |
| Chlorpromazine | | 76 mg/day | [25] |
| Trifluoperazine | | 17 mg/day | [25] |
| Perphenazine | | 16 mg/day | [25] |
| Fluphenazine | | 10 mg/day | [25] |
| Haloperidone | | 10 mg/day | [25] |
| **Reference price** | | | |
| Generic risperidone 2 mg | | 4 baht/day | [32] |
| Olanzapine 10 mg | | 189 baht/day | [33]^1^ |
| Clozapine 100 mg | | 3.5 baht/day | [33]^2^ |
| Chlorpromazine 100 mg | | 0.4 baht/day | [34] |
| Trifluoperazine 2 mg | | 1.0 baht/day | [33] |
| Perphenazine 8 mg | | 1.0 baht/day | [33] |
| Fluphenazine 2.5 mg | | 3.4 baht/day | [34] |
| Haloperidone 2 mg | | 2.5 baht/day | [33] |
| Average price of typicals | | 1.7 baht/day | [33-34]^3^ |
| Benzhexol 15 mg | | 1.14 baht/day | [34] |
| **Relative risk of EPS** | |  |  |
| Clozapine | | 0.17 | [26] |
| Olanzapine | | 0.39 | [26] |
| Risperidone | | 0.61 | [26] |
| **Blood test cost**  Laboratory | | 88 baht/time | [35] |
| Professional ’s time | | 42,863 | [27] |
| Nonprofessional ’s time | | 14,095 | [27] |
| Blood tests for the first year | | 26 time/year | [36] |
| Blood tests for the other year | | 12 time/year | [36] |
| **Hospital cost** | | | |
| Relative risk of relapse for atypicals vs. typicals | | 0.65 | [28] |
| Admission rate among people on typicals | | 0.27 | [25] |
| Reduction in hospitalization due to family interventions | | 3% | [18, 28]^4^ |
| **Patient and family cost for outpatient visit** | | | |
| Time cost of patient (25% of personal income) | | 9 baht/hour | [37] |
| Time cost of carer (25% of personal income) | | 9 baht/hour | [37] |
| Travel cost | | 217 baht/time | [25] |
| Time spent on hospital visit | | 5 hour /visit | [25] |
| % of people visiting hospitals with carers | | 39% | [25] |
| Outpatient visit | | 4 time/year | [31] |
| **Cost elements related to family interventions** | |  |  |
| Development and training cost for family interventions | |  |  |
| Accommodation | | 1,200 baht/night | Assumed based on the rate used in our survey [25] |
| Transportation | | 3,000 baht/person |
| Time cost for a training consultant | | 800 baht/hour |
| Daily allowance for a psychiatrist | | 1,000 baht/day |
| Allowance for a nurse | | 500 baht/day |
| Daily lunch and break | | 300 baht/person |
| Meeting room rental | | 400 baht/day |

^1)^ Based on data from Eli Lilly company

^2)^ Average cost computed based on various local pharmaceutical companies

^3)^ A weighted mean was used to measure the average costs of typicals: using the frequency of prescriptions for chlorpromazine, haloperidol, fluphenazine, perphenazine, trifluoperazine, which were the most common drugs used in 2008 [25]. Costs of typicals were estimated using the reference price of each drug from the Government Pharmaceutical Organization (GPO)

^4)^ Reduction in hospitalization rate from family intervention over and above the reduction in hospitalization from risperidone alone

**References**
